# New insights into rice phenology: discovering the effect of insolation on heading response

**DOI:** 10.1111/ppl.70132

**Published:** 2025-02-19

**Authors:** Ju‐Hee Kim, So‐Hye Jo, Ji‐Hyeon Moon, Seo‐Yeong Yang, Jae‐Kyeong Baek, Yeong‐Seo Song, Ji‐Young Shon, Hyeon‐Seok Lee

**Affiliations:** ^1^ Crop Production & Physiology Division, National Institute of Crop Science, Rural Development Administration Wanju‐Gun Republic of Korea

## Abstract

Precise growth management is required for climate‐smart and sustainable crop production in response to climate change, with the heading stage being the most important. Research on the control of heading in rice (*Oryza sativa*) has mainly focused on day length and temperature; however, research on the effects of insolation is limited. Therefore, this study analyzed the differences in rice growth and heading responses under different light intensity and temperature conditions. Five early‐maturing and seven medium–late‐maturing rice varieties were used for each japonica heading ecology type. Our results showed that leaf age development, an indirect measure of rice phenological development, was inhibited under low light intensity and low‐temperature conditions. Accordingly, the heading date was also delayed by approximately 18 days at low temperatures and 21 days at low light intensity, with no difference among ecotypes. We also found an interaction between temperature and light intensity, with the light intensity‐mediated delay in heading date being affected more by high temperatures. This study demonstrated that light intensity and temperature have a major effect on heading date variation, suggesting that the impact of insolation must be considered for the accurate prediction of heading stage variation. These results could shed new light on rice phenology research and contribute to the implementation of precision agriculture.

## 1 INTRODUCTION

Ceccarelli et al. (2010) predicted that rice production would decline by up to 41% by the end of the 21st century owing to climate change (Ceccarelli et al., 2010; Wheeler & Von Braun, 2013; Carrijo et al., 2017). At COP28, a roadmap for climate‐smart crop production was developed to increase rice productivity by 1.5% per year while limiting the temperature increase due to global warming to 1.5°C, with the goal of “zero hunger,” which is the target of Sustainable Development Goal 2 announced by the United Nations in 2015 (Acen et al., 2024). This requires precision agriculture to increase production efficiency while reducing chemical fertilizer use (Pande & Moharir, 2023). Predicting climate‐induced heading variability is essential for implementing precision agriculture in rice production. This is because most agricultural operations, such as fertilization and water management, are scheduled based on the heading date (Lee et al., 2019; Wei et al., 2020).

The heading stage, a major growth stage, is influenced by exogenous factors, including photoperiod, temperature, and nutrient availability (e.g., nitrogen treatment), among which photoperiod is a key factor (Liu et al., 2019; Wei et al., 2020). The growth stages of rice are divided into the photosensitive period (PSP), which is sensitive to day length, and the basic vegetative (BVP) and post‐photosensitive periods (PPP), which are sensitive only to temperature (Collinson et al., 1992; Mimoto et al., 1989). Studies on the mechanisms of heading and floral induction in rice have mainly been conducted during the PSP stage. Studies of the florigenic response in rice at the molecular and transcriptomic levels have been ongoing since Tamaki et al. (2007) published a study wherein Hd3a was speculated to be a florigen. Since then, various studies have been conducted on the mechanisms of the photosensitization response by identifying up‐ and downstream genes involved in mechanisms ranging from the photosensitization signal PhyB (photoreception) to MADS14 and 15 (panicle formation) depending on light wavelength and day‐length conditions, and more recently, the mechanisms of photosensitization response regulation using CRISPR‐cas9 (Shim and Jang, 2020; Zhou et al., 2021).

However, the mechanisms underlying rice heading response to temperature and insolation are poorly understood (Song et al., 2012). To date, the expression of major heading response genes, such as Hd3a and RFT1, has been reported to be increased at high temperatures, and a delayed photosensitization response under low‐temperature conditions related to the Hd1‐Ghd7‐Ehd1‐RFT1 pathway has been confirmed only under long‐day conditions (Luan et al., 2009; Nagalla et al., 2021). In addition, studies on insolation‐dependent heading and floral induction in rice have shown a delay in emergence as a function of insolation intensity and treatment time during night tides (Yin et al., 1997). Nevertheless, other insolation‐dependent studies are lacking.

Climate change has increased the average temperature, insolation changes, and atmosphere drying (Zandalinas et al., 2021). Therefore, for climate‐smart crop production in response to climate change, studying the response of heading to temperature and insolation conditions, which are predicted to fluctuate significantly in the future owing to climate change, is essential (Fedorov, 2022). Therefore, in this study, we aimed to characterize the growth and heading responses to temperature and irradiance in rice and provide a new physiological perspective on heading regulation.

## 2 MATERIALS AND METHODS

### 2.1 Plant materials and experimental design

The selected rice varieties used in this study are listed in Table S1. The rice varieties used in this study were selected to include major cultivated varieties with diverse levels of yield stability. The criteria of yield stability evaluated by the WAASBY results (Lee et al., 2023), a comparison of the coefficient of variation of grain yield in 300 rice varieties genetically analyzed by the Breeding Division of the National Institute of Crop Science (Lee et al., 2023). All cultivars were japonica‐type rice, and five early‐ and seven medium–late‐maturing cultivars were used for each ecotype (Table S1). Additional information on cultivar selection can be found in Table S1. Experimental varieties were provided by the Breeding Division of the National Institute of Crop Science.

To investigate the effects of temperature and light intensity on the yield of different rice genetic resources, the plants were grown in a growth chamber (Figure S1). with precisely adjustable temperature, day length, and light intensity at the artificial weather facility of the National Institute of Crop Science in Jeonju, South Korea (35°49′19″N, 127°8′56″E). Seedlings for transplantation were grown in nursery trays under controlled conditions of 28 ± 5°C and a light intensity of 700 μmol m^−2^ s^−1^ photosynthetically active radiation (PAR) for 7 days. All cultivars were transplanted with three plants per 1/5000a Wagner pots (3958 mL). A composite slow‐release fertilizer was applied at a rate of 9 kg/10a nitrogen, 4.5 kg/10a phosphate, and 5.7 kg/10a potassium, with an area ratio of three plants (planting distance: 30 × 14 cm). Temperature and light intensity treatments were initiated after an adaptation period of approximately 7 days after transplantation.

The experimental conditions included two temperature levels: high temperature (28 ± 5°C) and low temperature (22 ± 5°C), and two light intensity levels: control light intensity at 700 PAR (100%) and low light intensity at 160 PAR (20%). Based on previous studies (Lee et al., 2019; Lee et al., 2021), two temperature conditions were selected: 28 ± 5°C, where heading date is accelerated, and 22 ± 5°C, where heading date is delayed. The low light intensity condition was designed to simulate approximately 80% shading, representing the level of light reduction on cloudy days, based on findings from previous studies (Okawa et al., 2003; Choi et al., 2012). These conditions were combined to create four treatment groups: (1) high temperature + control light intensity (HT), (2) high temperature + low light intensity (HTLL), (3) low temperature + control light intensity (LT), and (4) low temperature + low light intensity (LTLL). The day length was set to 14 h (lights on: 07:00; lights off: 21:00). Temperature fluctuated hourly, with the lowest temperature set at 05:00 and the highest temperature at 14:00. The day length was set to 14 h (07:00 on, and 21:00 off ).

### 2.2 Growth investigation

Growth was monitored according to treatment conditions and cultivars for seven weeks at seven‐day intervals after transplantation. Leaf age, an indicator of vegetative development, was calculated using the following equation:

$$\text{Leaf}\;\mathrm{age}=\left(n-1\right)+\left(m÷M\right)$$

where *n* is the total number of leaves, including incomplete leaves, *M* is the length of the second fully expanded leaf from the top, and *m* is the length of the unexpanded leaf derived from the second leaf sheath. Leaf age is an indirect indicator to assess the transition among growth stages (Lee et al., 2021).

Tillers were assessed at the maximum tillering stage, 30 days before the heading date. The number of tillers was defined as those with at least two fully expanded leaves at the time of tiller measurement. Leaf width was measured at the central part of the second fully expanded leaf. The heading stage was defined as the number of days from the transplantation date until panicle emergence from the leaf sheath. The heading date is when 40–50% of the tillers have panicles. Culm length was measured from the ground to the neck of the panicle when all the panicles were removed after heading.

### 2.3 Statistical analysis

Statistical analysis was performed in SPSS (version 26; SPSS Inc.) and R studio (version 4.3.1; R Foundation for Statistical Computing) using a one‐way analysis of variance (ANOVA), followed by Duncan's multiple range test to test for significant differences at *p* < 0.05. The following equation (F) was used to model the development of leaf age based on the number of growing days after transplantation (Black and Leff, 1983). To quantify the rate of leaf age development (rF), a logistic model was applied to describe changes in leaf age over time:

$$F=\frac{\text{Lmax}}{1+e^{-\left(t-\mathrm{tm}\right)*\mathrm{rF}}}$$

Where *F* represents leaf age at time *t* (number of days after transplanting), *L*_*max*_ is the final leaf age, *r*_*F*_ is the rate of leaf age development until the final leaf age, and the time at which half of the final leaf age is reached (*t*_*m*_). *t*_*m*_ is also an indicator of the duration of development, as it refers to the point at which the development rate of leaf age is maximum. The values are coefficients determined by nonlinear regression analysis using SigmaPlot 10.0 (Grafiti LLC).

## 3 RESULTS

### 3.1 Effect of temperature and light intensity on rice growth and development

The logistic model analysis using time‐series data of leaf age is presented in Table 1 and Figure S2. Differences in the rate of leaf development (*r*_*F*_) under temperature and light intensity conditions—high temperature with control light intensity (HT), high temperature with low light intensity (HTLL), low temperature with control light intensity (LT), and low temperature with low light intensity (LTLL)—showed a decrease with lower light intensity and temperature. For early‐maturing cultivars, the *r*_*F*_ values were 0.073, 0.043, 0.049, and 0.035, respectively, while for medium‐late maturing cultivars, they were 0.065, 0.047, 0.040, and 0.032, respectively. Under HT conditions, low light intensity reduced *r*_*F*_ by 1.70‐fold and 1.38‐fold for early‐ and medium‐late maturing cultivars, respectively (Table 1; Figure S2). Under LT conditions, low light intensity reduced *r*_*F*_ by 1.40‐ and 1.25‐fold for early‐ and medium‐late maturing cultivars, respectively. Compared to HT conditions, LT conditions decreased *r*_*F*_ by 1.49‐ and 1.63‐fold for early‐ and medium‐late maturing cultivars, respectively (Table 1; Figure S2).

**TABLE 1:** Parameters of the logistic function used to describe rice leaf age by days after transplanting according to temperature and light intensity treatment from sowing to heading date.

Heading ecology type	Treatment	Development of leaf age Mean (SEM)			
		*L*_*max*_^†^	*r*_*F*_^††^	*t*_*m*_^†††^	*R* ^2††††^
Early maturing rice	HT	14.02 (0.06)	0.073 (0.002)	14.75 (0.34)	0.99
	HTLL	16.80 (0.20)	0.043 (0.001)	27.24 (0.81)	0.99
	LT	13.44 (0.13)	0.049 (0.002)	17.88 (0.67)	0.99
	LTLL	14.90 (0.22)	0.035 (0.001)	27.14 (1.02)	0.99
Medium–late maturing rice	HT	15.45 (0.08)	0.065 (0.002)	16.68 (0.41)	0.99
	HTLL	17.44 (0.15)	0.047 (0.002)	26.71 (0.57)	0.99
	LT	15.43 (0.20)	0.040 (0.002)	21.44 (0.89)	0.99
	LTLL	16.80 (0.30)	0.032 (0.001)	31.42 (1.29)	0.99

HTLL: High temperature, Low light; HT: High temperature; LTLL: Low temperature, Low light; LT: Low temperature. ^†^ *L*_*max*_ is the final number of leaves. ^††^ *r*_*F*_: rate of development until the final number of plants. ^†††^ *t*_*m*_: the time at which half of the final leaf age is reached. ^††††^ *R* ^2^: coefficient of determination.

Differences in the time point at which half the number of final leaves (*t*_*m*_) developed under temperature and light conditions showed delays with reduced light intensity and temperature (Table 1; Figure S2). For early‐maturing cultivars, *t*_*m*_ values were 14.75, 27.24, 17.88, and 27.14 under HT, HTLL, LT, and LTLL conditions, respectively, while for medium‐late maturing cultivars, they were 16.68, 26.71, 21.44, and 31.42, respectively. Low light intensity caused delays of 12.5 days and 10.0 days in *t*_*m*_ for early‐ and medium‐late maturing cultivars, respectively, under HT conditions, and 9.3 days and 10.0 days, respectively, under LT conditions. Comparatively, LT conditions delayed *t*_*m*_ by 3.1 days and 4.8 days for early‐ and medium‐late maturing cultivars, respectively, compared to HT conditions (Table 1; Figure S2).

Differences in the final leaf age (*L*_*max*_) under temperature and light conditions were as follows: For early‐maturing cultivars, *L*_*max*_ values were 14.02, 16.80, 13.44, and 14.90 under HT, HTLL, LT, and LTLL conditions, respectively. For medium‐late maturing cultivars, the corresponding *L*_*max*_ values were 15.45, 17.44, 15.43, and 16.80 (Table 1; Figure S2).

The development of leaf age, an indirect indicator of vegetative growth, showed a reduction in *r*_*F*_ and a delay in *t*_*m*_ under low light intensity and low temperature conditions. The reduction in *r*_*F*_ was more pronounced under low temperature, while the delay in *t*_*m*_ was greater under low light intensity. Additionally, *L*_*max*_ increased under low light intensity but decreased under low temperature (Table 1; Figure S2). The development of leaf age across cultivars and treatments is presented in Figure S3.

**FIGURE 1:**
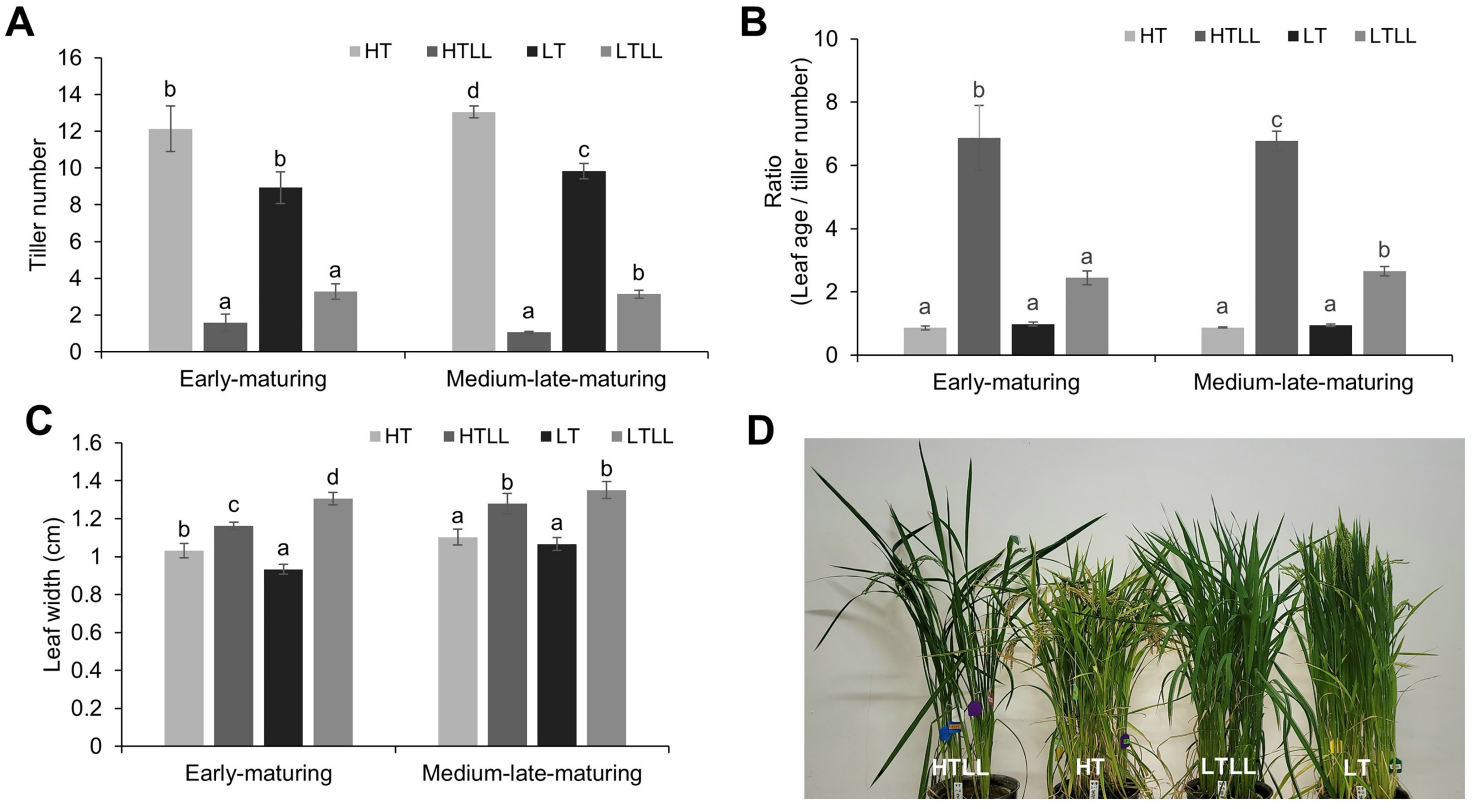
Morphological and physiological responses of rice under varying temperature and light intensity conditions. (A) Number of tillers 28 days after transplanting. (B) The ratio of the leaf age and tillers at 28 days after transplantation. (C) Leaf width of two rice ecotypes 89 days after transplanting. (D) Morphological changes under temperature and light intensity treatments in Samkwang (medium‐late maturing rice) at 28 days after transplantation. Data represents the mean ± SEM. Early‐maturing plants (*n* = 45) include 5 cultivars with 9 plants per cultivar. Medium–late‐maturing plants (*n* = 63) include 7 cultivars with 9 plants per cultivar. Data were analyzed using one‐way ANOVA. Means with different letters represent significant differences (*p* < 0.05) according to Scheffe's test.

Tiller number decreased under low light intensity and low temperature treatments, with the following trend: HT > LT > LTLL > HTLL. For early‐maturing cultivars, the values were 12.1 > 8.9 > 3.3 > 1.6, and for medium‐late maturing cultivars, they were 13.0 > 9.8 > 3.1 > 1.1 (Figure 1A). Under HT conditions, low light intensity reduced tiller number by 10.5 and 11.9 for early‐ and medium‐late maturing cultivars, respectively. Under LT conditions, the reduction was 5.6 and 6.7 for early‐ and medium‐late maturing cultivars, respectively (Figure 1A). Compared to HT conditions, LT conditions decreased tiller number by 3.2 in both early‐ and medium‐late maturing cultivars, indicating that light intensity had a greater impact on tiller reduction than temperature (Figure 1A).

Accordingly, the leaf age to tiller number ratio showed an opposite trend: HTLL > LTLL > LT > HT. For early‐maturing cultivars, the ratios were 6.87 > 2.44 > 0.98 > 0.89, and for medium‐late maturing cultivars, they were 6.77 > 2.65 > 0.94 > 0.87 (Figure 1B). These results suggest that low light intensity decreases tiller number while promoting the development of leaves on the main stem (Figure 1B). In line with these findings, the leaf width of the main stem also increased under low light intensity, with a greater increase observed under LT conditions compared to HT conditions (Figure 1C). These phenotypic differences are shown in Figure 1D. The differences in tiller number across cultivars and treatments are presented in Figure S4, and the differences in leaf width are shown in Figure S5.

### 3.2 Variations in days to heading under temperature and light intensity

**FIGURE 2:**
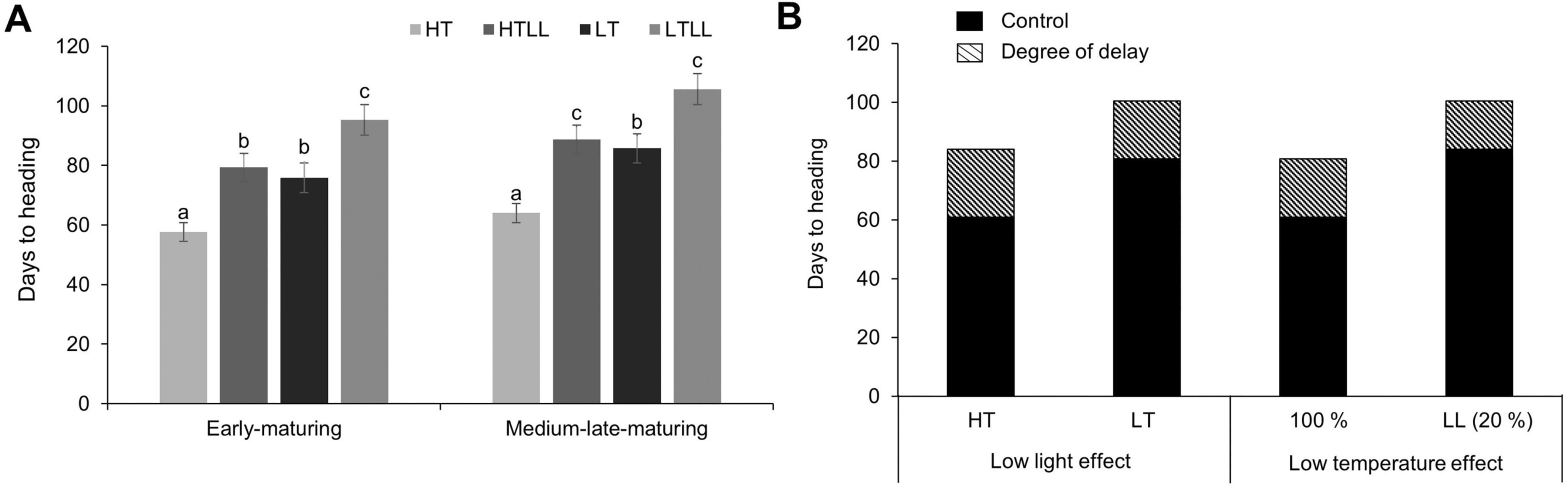
Differences in days to heading according to temperature and light intensity. (A) Differences among rice ecotypes. (B) Effect of temperature and light intensity on rice flowering (average of two types of values). The black‐filled portion of the bar graph is the number of days to heading under 100% light intensity in the low light effect section, and the number of days to heading under HT temperature in the low temperature effect section. The crossed‐out portion of the bar graph and the number are the number of days the heading was delayed by treatment. Data represents the mean ± SEM. Early‐maturing plants (n = 45) include 5 cultivars with 9 plants per cultivar. Medium–late‐maturing plants (n = 63) include 7 cultivars with 9 plants per cultivar. Data were analyzed using one‐way ANOVA. Means with different letters represent significant differences (p < 0.05) according to Scheffe's test. HTLL: High temperature, Low light; HT: High temperature; LTLL: Low temperature, Low light; LT: Low temperature.

Days to heading (DTH), similar to leaf development, was delayed under low light intensity and low temperature conditions. Under HT, HTLL, LT, and LTLL conditions, DTH values for early‐maturing cultivars were 57.69, 79.33, 75.82, and 95.29 days, respectively, while for medium‐late maturing cultivars, the values were 64.00, 88.78, 85.74, and 105.61 days, respectively (Figure 2A). Low light intensity under HT conditions delayed DTH by 21.64 and 24.78 days for early‐ and medium‐late maturing cultivars, respectively. Similarly, low light intensity delayed DTH by 18.13 and 21.74 days under LT conditions for early‐ and medium‐late maturing cultivars, respectively (Figure 2A).

Low light intensity, when averaged across maturing types, delayed DTH by 23.22 and 19.67 days under HT and LT conditions, respectively. Low temperature delayed DTH by 19.94 and 16.39 days under control light intensity and low light intensity conditions, respectively (Figure 2B). These results indicate that the impact of low light intensity was greater under HT conditions compared to LT conditions (Figure S6A), and the effect of low temperature was more pronounced under low light intensity conditions compared to control light intensity conditions (Figure S6B). The variation in DTH across treatments for each cultivar is presented in Figure S7.

Meanwhile, culm length, panicle length, and panicle number under each treatment are summarized in Table 2. Analysis of variance showed that differences in culm and panicle length due to light intensity were significant at the *p* < 0.01*p* < 0.01 *p* < 0.01 level, with a slight increase in length observed under low light intensity, while no significant differences were detected for temperature (Table 2). For panicle number, significant differences were observed due to both light intensity and temperature (*p* < 0.001 *p* < 0.001 *p* < 0.001), as well as their interaction (*p* < 0.01 *p* < 0.01 *p* < 0.01). Panicle number decreased under low light intensity but increased under low temperature (Table 2). Furthermore, the reduction in panicle number caused by low light intensity was more pronounced under HT conditions, while the increase due to low temperature was more pronounced under low light intensity conditions (LL) (Table 2).

**TABLE 2:** Changes in culm and panicle length and the number of panicles according to temperature and light intensity treatment from sowing to heading date.

Ecotype	Treatment	Culm length (cm)	Panicle length (cm)	Panicle number (ea)
Early‐maturing	HT	57.2 ± 2.2 a	19.6 ± 0.4 a	11.1 ± 0.6 a
	HTLL	62.9 ± 5.1 a	19.4 ± 0.7 a	2.7 ± 0.4 b
	LT	54.6 ± 2.4 a	17.4 ± 0.2 a	12.2 ± 0.6 a
	LTLL	66.4 ± 5.1 a	19.5 ± 0.9 a	4.5 ± 0.8 b
	Average	60.3	19.0	7.6
Medium–late‐maturing	HT	56.7 ± 2.7 ab	17.6 ± 0.5 a	11.7 ± 0.5 a
	HTLL	56.4 ± 0.7 ab	18.8 ± 1.0 a	2.1 ± 0.2 c
	LT	51.2 ± 3.8 b	17.0 ± 0.6 a	12.3 ± 0.6 a
	LTLL	61.2 ± 2.7 a	17.2 ± 0.6 a	5.1 ± 0.4 b
	Average	56.4	17.6	7.8
**Analysis of variance**				
Ecotype (E)		ns	**	ns
Light intensity (L)		**	**	***
Temperature (T)		ns	ns	***
Interaction (E*L)		ns	ns	ns
Interaction (E*T)		ns	ns	ns
Interaction (L*T)		*	ns	**
Interaction (E*T*L)		ns	ns	ns

HTLL: High temperature, low light intensity; HT: High temperature; LTLL: low temperature, low light intensity; LT: Low temperature. ns: non‐significant (*p* > 0.05), *, **, ***: significant at *p* < 0.05, 0.01, and 0.001, respectively. Means followed by different letters represent significant differences (*p* < 0.05) according to Duncan's test.

## 4 DISCUSSION

Studies on plant responses to light in various species have primarily focused on the presence or absence of light and light quality (Smith, 1982; Ballaré and Pierik, 2017). In some horticultural crops, similar to the results of this study, reduced light intensity has been reported to delay flowering (Sedgley and Buttrose, 1978; Rezazadeh et al., 2018). However, research into the regulation of plant development by light intensity is still in its early stages (Xu et al., 2021). In the model plant Arabidopsis thaliana, recent studies have shown that low light (LL) conditions increase the expression of miR156/miR157, thereby prolonging the juvenile vegetative phase (Xu et al., 2021). In rice, studies on the effects of light intensity on development are highly limited. The few reported findings include delayed heading when light was applied at night to interrupt the photoperiod response (Yin et al., 1997).

Recently, most rice modelling research that simulated rice growth rarely considered phenology, and research on phenology in this area is stagnant (Van Oort et al., 2011; Chen et al., 2020). This is because when phenology prediction errors occur, they are poorly interpreted (Van Oort et al., 2011; Chen et al., 2020). Most rice growth models used to predict the heading stage also predict the developmental stage based on the effects of day length and temperature from meteorological data (Egli and Bruening, 1992; Wang and Engel, 1998; Chen et al., 2020). However, the effect of light intensity on rice heading has not yet been extensively analyzed (Yin et al., 1997).

Owing to the reduced rate of leaf emergence rate during the transition from vegetative to reproductive growth (Collinson et al., 1992; Mimoto et al., 1989; Lee et al., 2021), leaf age development has been used as an indirect indicator for assessing growth phase transitions. In this study, we observed a deceleration in leaf age development in the main stem under low light and low temperature conditions, along with a delay in the heading date (Figure 1, Table S2, Figure S6). These findings indicate that similar to temperature, the light intensity can significantly influence growth phase transitions and heading response. Eventually, if insolation is not considered, the error rate will increase, and with climate change, this will probably increase even more. This is because day length shows little variation at the same time and location, whereas temperature and insolation are highly variable (Van Oort et al., 2011; Lee et al., 2019).

Meanwhile, most rice growth and productivity assessments under climate change scenarios primarily consider the effects of temperature and CO₂ changes (Zafar et al., 2018; Li et al., 2024). Although some studies have addressed the influence of solar radiation or light intensity (Lee et al., 2013; Guo et al., 2024), research in this area remains insufficient. Based on the findings of this study, the effects of light intensity appear to be substantial, highlighting the need to integrate assumptions about light conditions into climate change scenarios that examine temperature and CO₂ impacts on productivity. For example, Lee et al. (2022) reported an unusual 4–5 days delay in heading under conditions where increased temperatures were expected to accelerate heading, attributing this delay to reduced solar radiation during a specific growth stage.

We believe that the inaccuracy in heading‐date prediction systems will diminish if the findings of the present study are incorporated into future crop models. Furthermore, these results offer a novel physiological climatic perspective that will be invaluable for studying phenological responses in rice. These results will provide a crucial foundation for implementing precision agriculture. Incorporating insolation conditions into crop prediction models is believed to significantly enhance rice productivity and efficiency, potentially facilitating sustainable agricultural development.

## AUTHOR CONTRIBUTIONS

**Ju‐Hee Kim**: First Author, Design of experiments, data analysis, and writing–original draft preparation. **Hyeon‐Seok Lee**: Co‐First Author and Corresponding Author, Conceptualization, supervision, design of experiments, data analysis, and writing–original draft preparation. **So‐Hye Jo; Ji‐Hyeon Moon; Seo‐Yeong Yang; Jae‐Kyeong Baek; Yeong‐Seo Song**: Data analysis. **Ji‐young Shon**: Supervision. All the authors discussed the results and contributed to this study.

## FUNDING INFORMATION

This work was supported by the Rural Development Administration National Research Project Project Name: Investigation of rice heading and ripening characteristics under abnormal weather (low light intensity, high temperature) [Project No. PJ017247].

## CONFLICT OF INTEREST STATEMENT

The authors declare no competing interests.

## Supporting information


**Appendix S1:** Supporting Information

## Data Availability

All data and analyses are presented in the manuscript or the Supporting Information. The source data for Figures 1, 2, Table 1, 2, Figures S1–S7, and Tables S1 are provided as Source Data files.
