# Supplementary material for: New insights into rice phenology: discovering the effect of insolation on heading response
Source: Physiol Plant. 2025 Feb 19;177(1):e70132. doi: 10.1111/ppl.70132 (PMC11836917; doi:10.1111/ppl.70132)
Supplement: Supplementary file 1 — Appendix S1: Supporting Information [file PPL-177-e70132-s001.docx]

**Short Communication**

**New insights into rice phenology: discovering the effect of insolation on heading response**

Ju-Hee Kim^a†^, So-Hye Jo^a^, Ji-Hyeon Moon^a^, Seo-Yeong Yang^a^, Jae-Kyeong Baek^a^, Yeong-Seo Song^a^, Ji-Young Shon^a^, Hyeon-Seok Lee^a†*^

^a^Crop Production & Physiology Division, National Institute of Crop Science, Rural Development Administration, Wanju-Gun 55365, Republic of Korea

†Ju-Hee Kim and Hyeon-Seok Lee contributed equally to this work

**^*^Corresponding author:** Hyeon-Seok Lee

This file contains 1 supplementary tables and 7 supplementary figures.

**Table S1.** List of selected rice varieties.

| Early maturing | 1. Unkwang G 034 (High yield stability)  2. Asemi 1 ho G 071 (Low yield stability)  3. Junamjosaeng G 042 (Medium yield stability)  4. Undoobyeo G 022 (Medium yield stability)  5. Odaebyeo G 002 (Medium yield stability) |
| --- | --- |
| Medium-late  maturing | 1. Dongin 1 ho G 192 (High yield stability)  2. Sukwang G 238 (High yield stability)  3. Saeilmi G 241 (High yield stability)  4. Nampyeongbyeo G 180 (Low yield stability)  5. Samkwang G 197 (Low yield stability)  6. Misomi G 247 (Low yield stability)  7. Deuraechan G 222 (Low yield stability) |


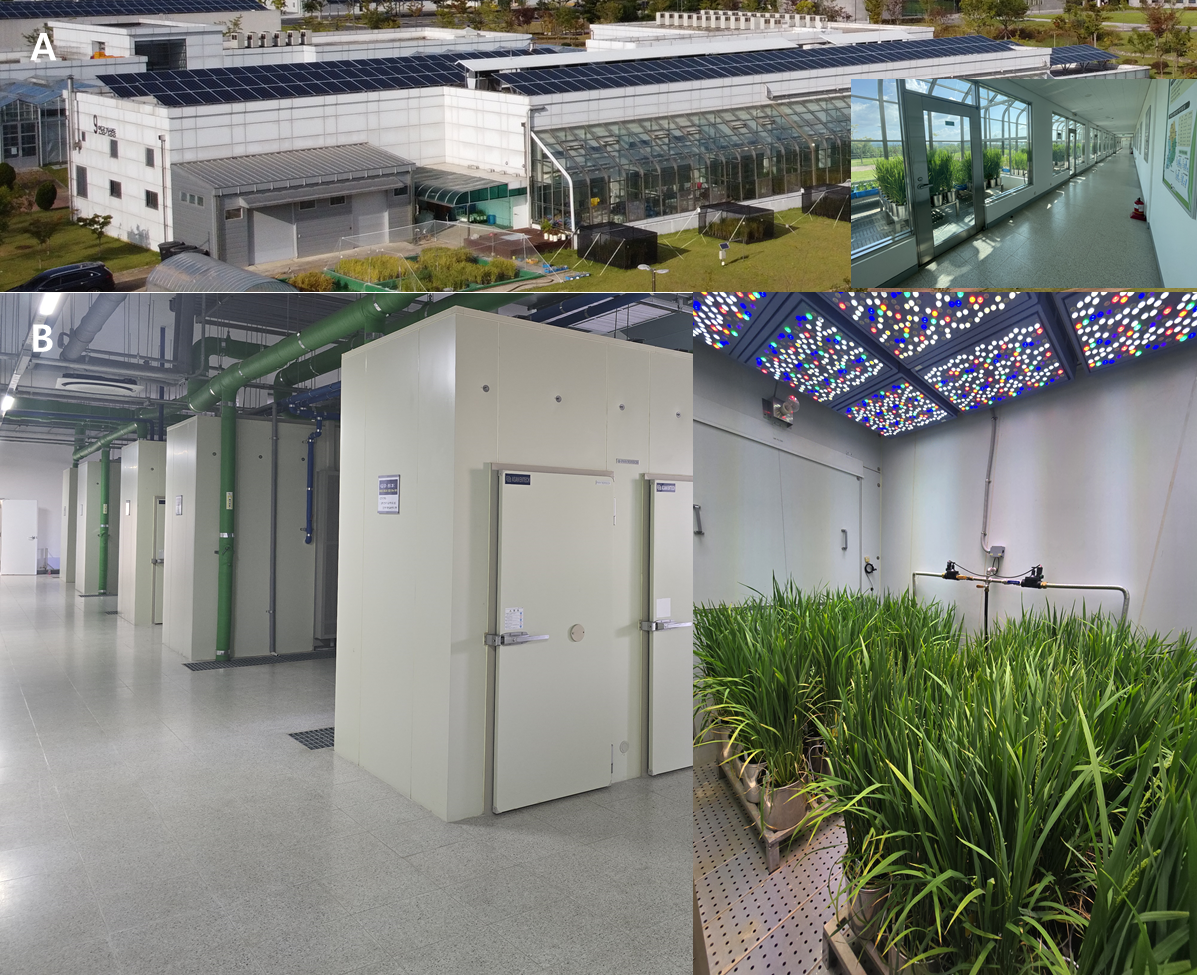


**Fig. S1. Controlled environment facility at the national institute of crop science (A), and lighting chamber where light intensity, temperature, and humidity can be artificially controlled (B).**


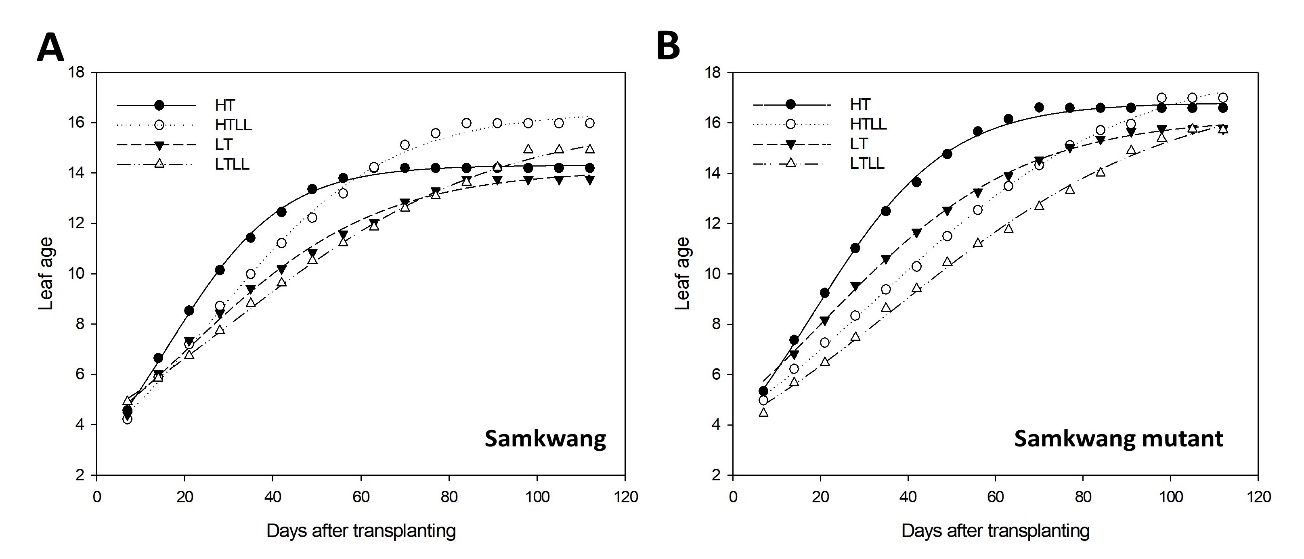
**Fig. S2. Effects of temperature and light intensity on leaf development by ecotype.** Logistic models of rice plant leaf age development after transplanting according to temperature and light intensity after transplanting to the heading date. (A) Leaf age of early-maturing plants (n=45, 5 cultivars with 9 plants per cultivar). (B) Leaf age of medium–late-maturing plants (n=63, 7 cultivars with 9 plants per cultivar). Data represents the mean of leaf age development.

**
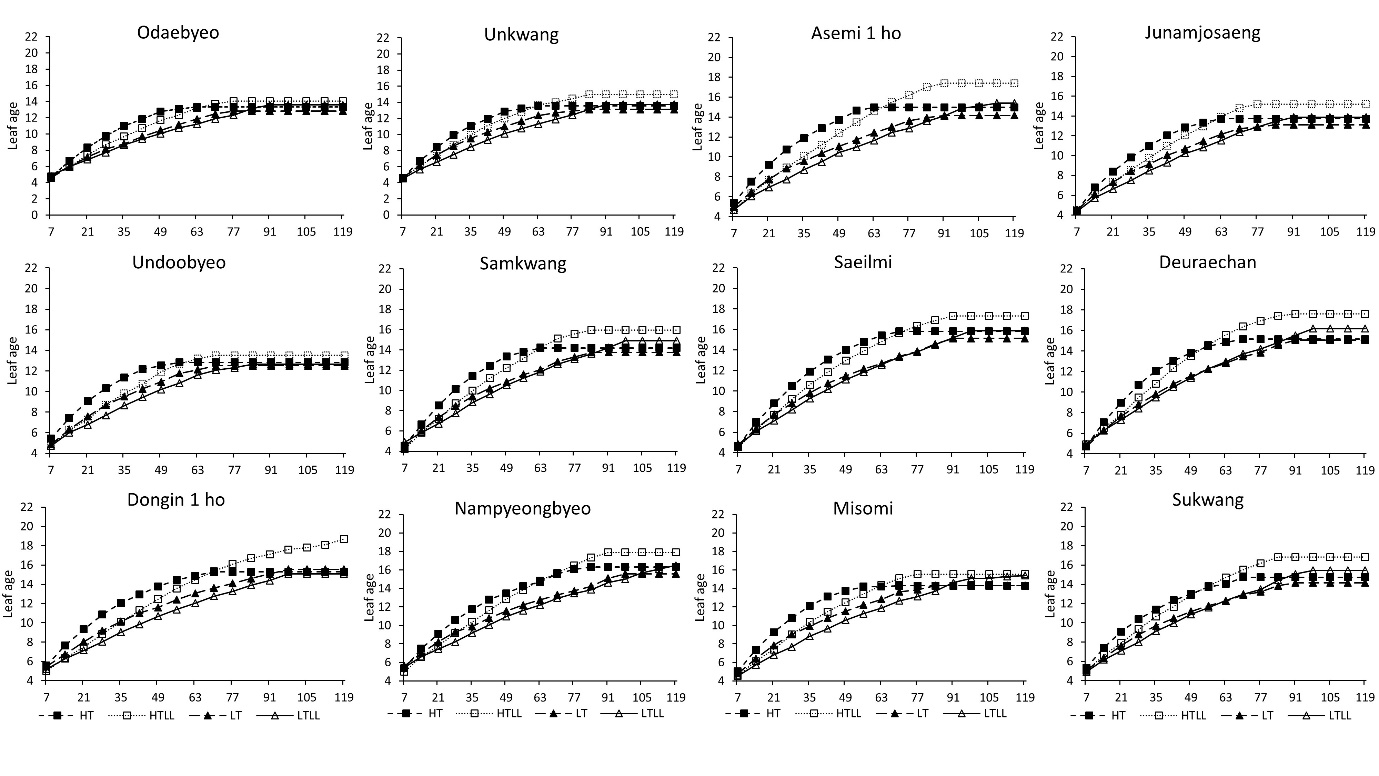
**

**Fig. S3. Differences in rice leaf development from transplanting to heading by variety according to temperature and light intensity.** Plants were grown under high temperature and low light intensity/HTLL (■), high temperature/HT (□), low temperature and low light intensity/LTLL (△), or low temperature/LT (▲) conditions in an artificial light growth room. Symbols show the average leaf age (n=9). Early maturing: ‘Odaebyeo,’ ‘Unkwang,’ ‘Asemi 1 ho,’ ‘Junamjosaeng,’ and ‘Undoobyeo’; Medium–late maturing: ‘Samkwang,’ ‘Saeilmi,’ ‘Deuraechan,’ ‘Dongin 1 ho,’ ‘Nampyeongbyeo,’ ‘Misomi,’ and ‘Sukwang.’


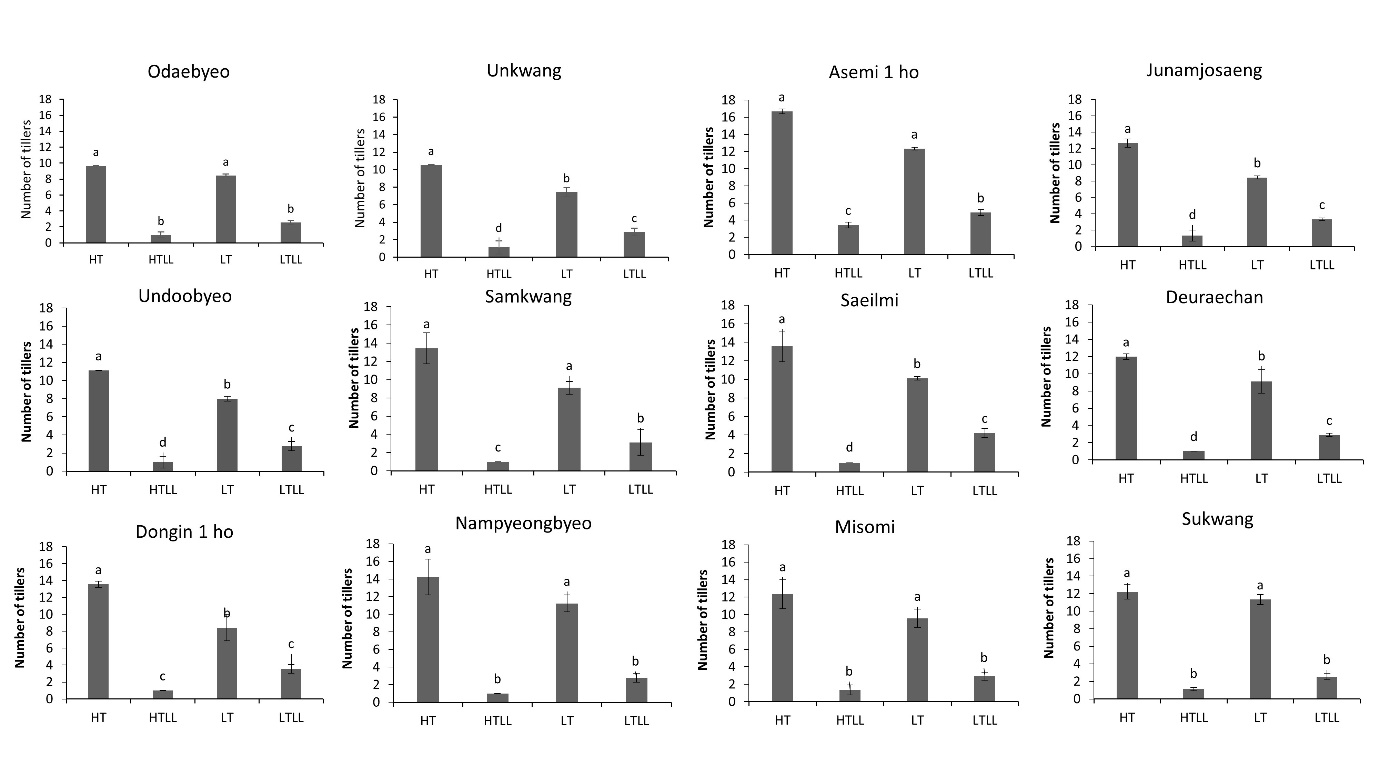
**Fig. S4. Differences in rice tiller development by variety according to temperature and light intensity.** The number of tillers was investigated 28 days after transplanting (n=9). Data represents the mean ± SEM. Data were analyzed by one-way ANOVA. Means with different letters represent significant differences (*p* < 0.05) according to Scheffe’s test. Early maturing: ‘Odaebyeo,’ ‘Unkwang,’ ‘Asemi 1 ho,’ ‘Junamjosaeng,’ and ‘Undoobyeo’; Medium–late maturing: ‘Samkwang,’ ‘Saeilmi,’ ‘Deuraechan,’ ‘Dongin 1 ho,’ ‘Nampyeongbyeo,’ ‘Misomi,’ and ‘Sukwang.’


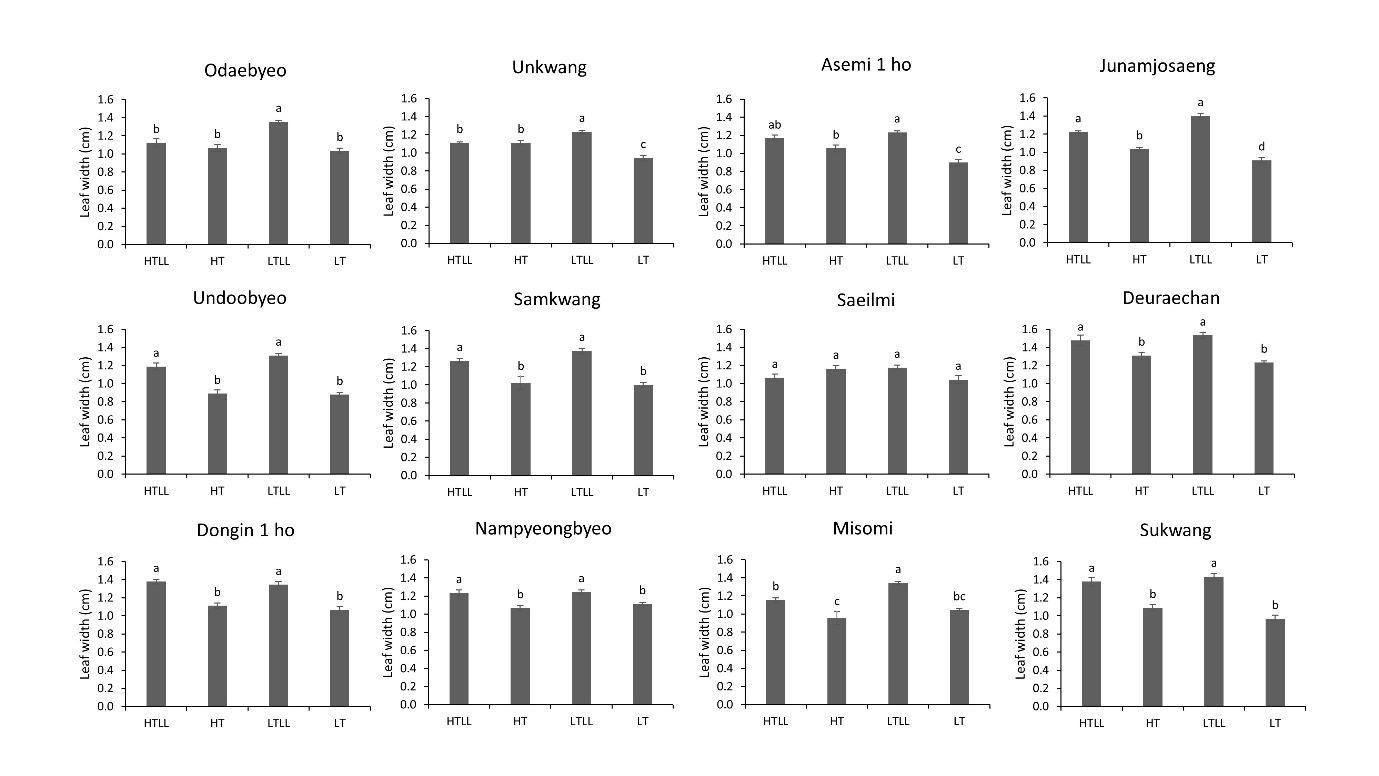


**Fig. S5. Differences in rice leaf width by variety according to temperature and light intensity.** Leaf width was investigated 89 days after transplanting. Data represents the mean ± SEM of independent replicates (n=9). Data were analyzed by one-way ANOVA. Means with different letters represent significant differences (*p* < 0.05) according to Scheffe’s test. Early maturing: ‘Odaebyeo,’ ‘Unkwang,’ ‘Asemi 1 ho,’ ‘Junamjosaeng,’ and ‘Undoobyeo’; Medium–late maturing: ‘Samkwang,’ ‘Saeilmi,’ ‘Deuraechan,’ ‘Dongin 1 ho,’ ‘Nampyeongbyeo,’ ‘Misomi,’ and ‘Sukwang.’


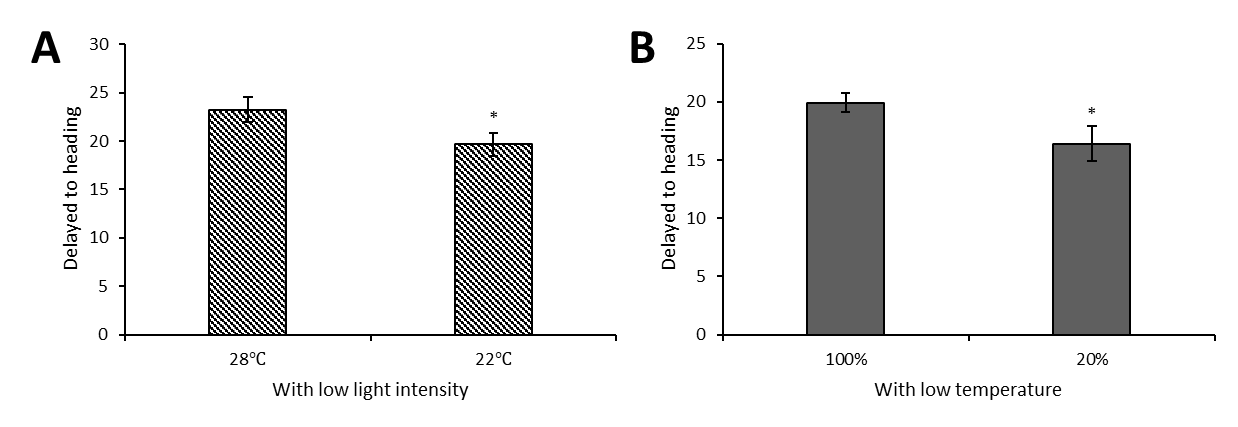


**Fig. S6. Effect of growing days from transplanting to heading according to temperature and light intensity.** (A) Effect of low light intensity under different temperature. (B) Effect of low temperature under different light intensity. Date represent the mean ± SE at *P* < 0.05(*) and *P* < 0.01(**).


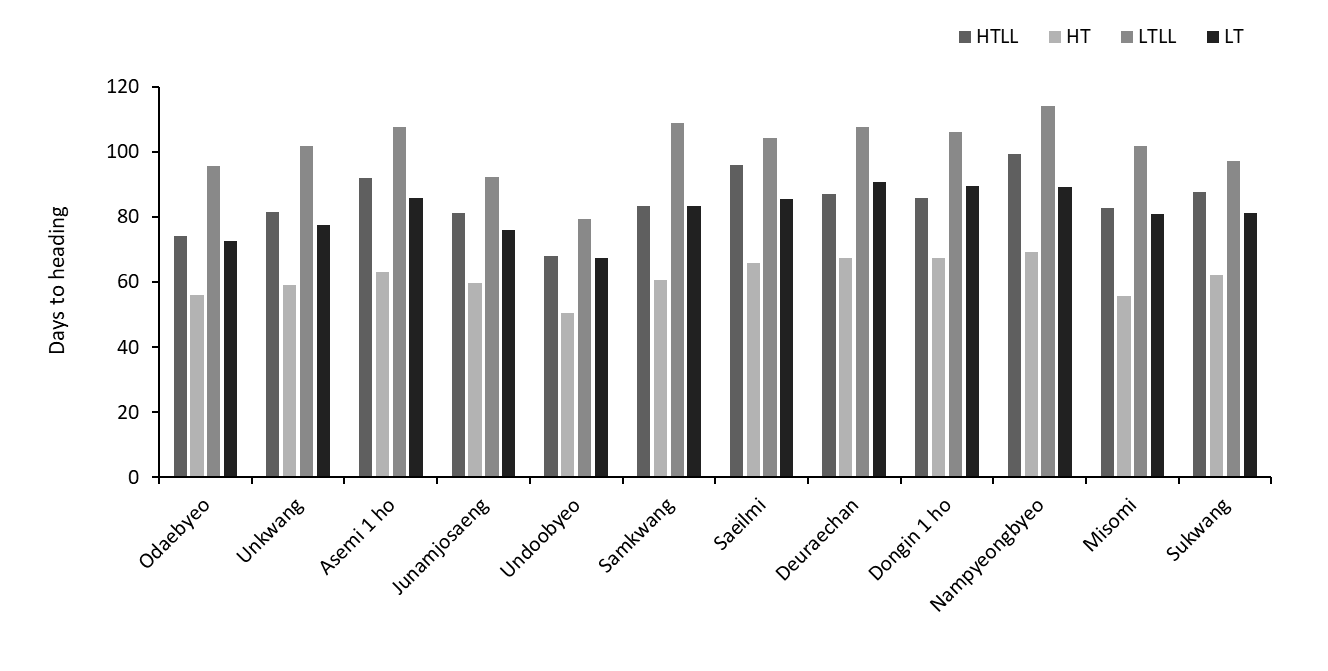


**Fig. S7. Differences in days to heading by variety according to temperature and light intensity.**
